# Zygapophysial joint blocks in chronic low back pain: a test of Revel's model as a screening test

**DOI:** 10.1186/1471-2474-5-43

**Published:** 2004-11-16

**Authors:** Mark Laslett, Birgitta Öberg, Charles N Aprill, Barry McDonald

**Affiliations:** 1 Department of Health and Society: Physiotherapy, Faculty of Health Sciences, Linköpings Universitet, SE-581581-85, Sweden; 2 Magnolia Diagnostics, 2718 Cadiz St, New Orleans LA 70115, LA, United States of America; 3 Massey University, Institute of Information and Mathematical Sciences, Albany, New Zealand

## Abstract

**Background:**

Only controlled blocks are capable of confirming the zygapophysial joints (ZJ) as the pain generator in LBP patients. However, previous workers have found that a cluster of clinical signs ("Revel's criteria"), may be valuable in predicting the results of an initial screening ZJ block. It was suggested that these clinical findings are unsuitable for diagnosis, but may be of value in selecting patients for diagnostic blocks of the lumbar ZJ's. To constitute evidence in favour of a clinical management strategy, these results need confirmation. This study evaluates the utility of 'Revel's criteria' as a screening tool for selection of chronic low back pain patients for controlled ZJ diagnostic blocks.

**Methods:**

This study utilized a prospective blinded concurrent reference standard related validity design. Consecutive chronic LBP patients completed pain drawings, psychosocial distress and disability questionnaires, received a clinical examination and lumbar zygapophysial blocks. Two reference standards were evaluated simultaneously: 1. 75% reduction of pain on a visual analogue scale (replication of previous work), and 2. abolition of the dominant or primary pain. Using "Revel's criteria" as predictors, logistic regression analyses were used to test the model. Estimates of sensitivity, specificity, predictive values and likelihood ratios for selected variables were calculated for the two proposed clinical strategies.

**Results:**

Earlier results were not replicated. Sensitivity of "Revel's criteria" was low sensitivity (<17%), and specificity high (approximately 90%). Absence of pain with cough or sneeze just reached significance (p = 0.05) within one model.

**Conclusions:**

"Revel's criteria" are unsuitable as a clinical screening test to select chronic LBP patients for initial ZJ blocks. However, the criteria may have use in identifying a small subset (11%) of patients likely to respond to the initial block (specificity 93%).

## Background

It is estimated that 15 – 40% of chronic low back pain patients have pain arising from the lumbar zygapophysial joints (ZJ)[1,2]. Previous studies have indicated that historical and physical examination findings cannot predict results from diagnostic ZJ blocks [1-6]. A specific sequence of injections of local anesthetic under fluoroscopic guidance into the joint space or targeting the medial branches of the dorsal rami, are reference standards for diagnosis. For the ZJ to be considered the sole source of pain, there must be a total or near total pain ablation for a time consistent with the known properties of the anesthetic agent[7,8]. In a recent study by Revel M et al (1998), the authors found that a cluster of seven items, (hereinafter called Revel's criteria) were shown to be of value in predicting a 75% reduction of pain following a single intra-articular anesthetic injection into the ZJs. The items in the cluster are: age over 65 years, pain well relieved by recumbency, no exacerbation of pain with: coughing and sneezing, forward flexion, extension, rising from flexion and the extension-rotation test[9]. These authors suggested two clinical strategies: Strategy 1 consists of five or more items being true. Strategy 2 is the same except that one of the true statements must be 'pain well relieved by recumbency'. Estimated sensitivities and specificities for strategies 1 and 2 were 100/92%, and 66/80% respectively. A subsequent study of 200 consecutive patients using double blocks failed to confirm these findings[6].

Because prognosis for acute low back is good, invasive and expensive and invasive diagnostic testing cannot be justified. However, persistent disabling pain needs more intensive investigation in order to determine appropriate management strategies. Back pain of ZJ origin may be treated using intra-articular steroid injection[7,10]., or radio frequency neurotomy [11-14]. However, the low prevalence of the condition dictates that a clinical selection process capable of identifying patients unlikely to respond to the initial screening diagnostic block is desirable. Patients with a low probability of a positive anesthetic response need not be subjected to the screening block and the tissue origin of pain should be sought elsewhere. Revel's criteria are currently the only documented clinical means by which response to an initial screening block may be predicted, but it remains a provisional finding only, until further research confirms the previous findings.

The current study objectives were to estimate the predictive value of the two clinical strategies of Revel et al (1998), using similar measurement parameters (75% reduction in pain VAS after ZJ block), and apply alternative analytic methods to explore any potential utility of the variables used.

## Methods

### Design

This study utilized a prospective, blinded, concurrent, reference standard-related validity design with intra-articular ZJ or medial branch blocks as the reference standard against which clinical variables were compared. Local Institutional Review Board approval was granted at the beginning of the study. A 75% or more reduction in pain following ZJ block on pain VAS was designated as reference standard A, the same standard used by Revel et al (1998). Based on pain drawings and patient self-report, complete abolition of the patient's primary or dominant pain was designated as reference standard B. Dominant pain location was acquired by pain drawing and direct questioning, and documented prior to clinical examination and diagnostic injection in a prospective manner. Post injection dominant pain location was acquired by reference to pain drawings and direct questioning also.

### Patients

Patients with low back pain with or without lower extremity symptoms, referred to a private radiology practice in New Orleans, USA specializing in the diagnosis of spinal pain, were invited to participate in the study. Patients receiving ZJ blocks were either referred specifically for that procedure or had the procedure included in their radiology examination based on pre-injection clinical evaluation by the injectionist (CA). Between May 2001 and October 2002, physical therapists attended the clinic in blocks that ranged from 4 to 8 weeks (ML) and examined patients. Normal scheduling was not affected by the presence of the visiting therapists, so patients were consecutive during these periods. All patients had undergone imaging studies prior to referral from a variety of medical and paramedical practitioners. Some were self-referred.

Patients were excluded from the study if they were unwilling to participate, were too frail to tolerate a physical examination, or were deemed by any member of the clinic team to be unable to comprehend the study procedure. Prior to the formal clinical examination, clinic staff recorded basic demographic and medical data.

### Measurements

#### Pain

100 mm visual analog scales (VAS) scales for current, best and worst pain. A current pain VAS was repeated after the clinical examination and following ZJ blocks. The 23-point Roland-Morris Disability Questionnaire [15] was completed to evaluate disability, and psychosocial distress estimated using the Zung Depression Index[16], the Modified Somatic Perception Questionnaire[17] and the Distress Risk Assessment Method (DRAM) [18].

### The physical examination

**Figure 1:**
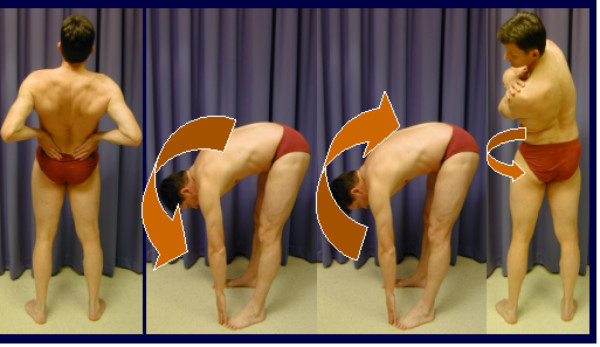
The four physical examination components of Revel's criteria for lumbar zygapophysial joint pain: standing flexion, returning from standing flexion, standing extension, the extension rotation test.

History taking and a structured physical examination were carried out by a physical therapist with 30 years of clinical experience as a manipulative therapist (ML). Some patients were examined by a physical therapist with 17 years experience (SBY). The clinical examination occupied 30 to 60 minutes and included many tests besides those necessary for the current analysis, as part of a larger project. Inconclusive findings or incomplete examinations were documented. The physical examination included a visual assessment of range of motion, recording anatomical location of dominant pain, nerve tension tests, key muscle strength tests, tendon reflex tests, light touch sensitivity, a McKenzie[19] styled examination and where possible, Waddell's tests for signs of inappropriate pain behaviour[20]. The data for Revel's criteria[9]were obtained in a prospective and systematic manner using standardized language and terminology. The four physical tests that form part of the criteria are depicted in Figure 1. (see file Figure 1 Revels criteria.png)

### Radiology examination

Prior to ZJ blocks, the radiologist reviewed case notes and imaging studies, and conducted a physical examination that guided the type of diagnostic procedure to be employed and the target structures. Intra-articular ZJ joint injection or MBB using standard technique[5] was carried out by an interventional radiologist (CA) with 20 years experience, or by an injectionist under his guidance. Patient pain responses to injections were recorded as 0.5 cc Lidocaine 2% was slowly injected into the target joint or at medial branch targets. Pain intensity 100 mm VAS's were recorded 30 to 45 minutes post procedure, then hourly in a pain journal for eight hours post-injection. Reference standards A and B were evaluated. A positive anesthetic response was recorded if reduction or abolition of pain lasted the known duration of lidocaine, about one and a half hours. Where appropriate and possible, positive responders were rescheduled for confirmatory blocks using bupivacaine 0.75%. A ZJ source of pain was confirmed if a confirmatory block was positive and relief of pain lasted for at least four hours. Some patients received ZJ blocks and sacroiliac joint injections during the same session. If the combined block was positive, the patient was scheduled to return for confirmatory blocks to identify which structure was responsible for the effect. If the combined block produced less than 75% reduction in pain, a negative ZJ block was recorded.

### Blinding

Physical therapists conducting the clinical examination were blinded to the results of previous imaging studies and diagnostic injections, the Roland, Zung and MSPQ questionnaires. The injectionist was blinded to the results of the physical therapy examination and diagnostic conclusions.

### Data analysis

Basic statistical values for demographic variables and regression analyses were calculated using statistics software (Minitab version 13.31 © Minitab Inc 2000). Differences between included and excluded patients were evaluated with the student's t, chi square, and Kruskal-Wallis tests where appropriate. Significance for differences was set at p < 0.05.

Calculations of sensitivity, specificity, predictive values and likelihood ratios with 95% confidence intervals were performed using Confidence Interval Analysis software © Bryant T.N. 2000[21].

## Results

Initial ZJ blocks were carried out on 151 chronic low back pain patients. Thirty-four patients were excluded from analysis as they received another intervention in the same procedure session and did not return for differentiating and confirmatory blocks. One case was excluded through incomplete data on Revel's criteria. Following ZJ block, 27 of 116 patients satisfied reference standard A. Data required for determination of Reference standard B were missing for five of the 116 cases. Eighteen of these 111 patients satisfied reference standard B. Table 1 contains demographic and other descriptive characteristics with comparisons between included and excluded patients. Included patients had longer time off work than excluded patients (mean 55 versus 99 weeks) but otherwise had similar characteristics.

In specifically evaluating Revel's criteria against reference standard A, logistic regression failed to achieve significance as a model (n = 108, p = 0.46). Two variables; "absence of pain with coughing and sneezing" and "no exacerbation of pain rising from flexion", showed a trend towards significance as predictors within the model (p = 0.07).

Using Revel's criteria within a logistic regression with reference standard B as the response variable, a strong trend towards significance was reached (n = 100, p = 0.06). One component variable (age over 65) individually reached significance within the model: (p = 0.004, odds ratio 16.1 with 95% confidence intervals of 2.4 and 107.8). In the whole sample 12.5% were aged over 65 years whereas of the 19 positive responders six were over 65 years (31.6%).

The same patients satisfied both strategies of Revel et al. Estimated sensitivity, specificity, predictive values and positive likelihood ratios for both strategies of Revel et al are presented in Table 2. With respect to reference standards A & B, sensitivities are low at 11 and 17%, specificities are high at 91 and 93% and likelihood ratios are 1.2 and 2.5 respectively.

Of the 27 patients satisfying reference standard A, 13 (48%) returned for confirmatory ZJ blocks. Three of these reported 75% or more reduction in pain following the confirmatory block. None of the three patients with confirmed ZJ pain satisfied Revel's criteria.

## Discussion

The current data produces results that are in stark contrast to those of Revel et al (1998) with sensitivity low and specificity high. However, 'no pain with cough and sneeze' and 'no exacerbation of pain rising from flexion' approached statistical significance in relation to a 75% reduction pain after ZJ block (reference standard A). These variables are in line with Revel's results. Age over 65 is associated with reference standard B (abolition of primary pain). Likelihood ratios for the criteria are lower in the current data also. Some of the differences in results may be explained by a number of factors:

1. Revel et al's study was a placebo controlled design whereas we did not routinely utilize a similar or equivalent control.

2. The patients in Revel et al's sample were older (mean 58 versus 43 years), and had a shorter duration of symptoms (mean 78 versus 160 weeks).

3. It is also possible that the high level of standardization when acquiring the criteria data in the current study, and the prospective methodology might have contributed to the differences in results.

Following anaesthetic blocks, patients frequently state that the pain prompting consultation is abolished, yet a post-procedure pain VAS still registers more than 0/100. In this study reference standard B was evaluated as an alternative to the usual standard, so that pain in areas above the lumbar spine or pain directly attributable to the needle insertion site do not result in inappropriate post procedure VAS scores. In the interests of developing more precise instruments documenting results of diagnostic blocks, we propose that in future studies, the VAS scale should specifically refer to the primary pain complaint for which the diagnostic block is undertaken.

Based on the current data, Revel's criteria are not suitable as a screening test to select patients suitable or unsuitable for an initial screening ZJ block. Such a screening device should have high sensitivity to ensure that most patients likely to respond are included in the initial diagnostic block. Our study suggests that, at best, Revel's criteria, might identify a small subset (11%) of patients likely to respond to a screening block (specificity 93%).

## Conclusions

Neither strategy utilizing Revel's criteria is suitable as a clinical screening device for selection of chronic LBP patients for initial diagnostic ZJ blocks. In contrast to Revel's findings, the current data demonstrated low sensitivity and high specificity for these clinical criteria. The high specificity of the criteria reported in this study relates to a single uncontrolled screening block. Consequently these criteria can not considered diagnostic of painful lumbar ZJ. Only placebo controlled or double ZJ blocks are able to diagnose this source of low back pain.

## Competing Interests

The author(s) declare that they have no competing interests.

## Authors' contributions

ML conceived the design of the study, examined all but 10 patients, carried out data analysis and prepared the manuscript

BÖ assisted in project design and manuscript preparation

CNA assisted in project design, provided facilities and conducted fluoroscopically guided injections

BMcD carried out data analysis and assisted in manuscript preparation.

All authors have read and approved the final manuscript.

## Pre-publication history

The pre-publication history for this paper can be accessed here:



## Figures and Tables

**Table 1:** Basic demographic data results for chronic LBP patients receiving screening ZJ blocks

	**All patients (n = 151)**			**Excluded patients (n = 35)**			**Included patients (n = 116)**			**Significance**
**Variable**	Mean	Median	STD	Mean	Median	STD	Mean	Median	STD	p value

Age (years)	44.3	43.0	13.2	48.03	48.0	14.3	43.2	41.5	12.6	0.09
Duration of current symptoms (weeks)	147.1	91.0	170.2	103.7	74.0	93.5	160.2	104.0	186.2	0.08
Time off work (weeks)	89.5 (n = 69)	74.0	82.4	55.4 (n = 15)	56.0	41.3	99.0 (n = 54)	82.0	88.6	0.04*
VAS (today)	55.9	61.0	24.6	50.8	53.0	27.0	57.4	62.0	23.7	0.24
VAS (at best)	30.3	27.0	22.5	24.6	15.0	22.5	32.0	30.5	22.3	0.06
VAS (at worst)	85.8	89.0	13.3	80.6	87.0	18.4	87.4	90.0	10.9	0.07
Roland Morris Questionnaire	17.8	19.0	4.8	18.0	19.0	4.6	17.7	19.0	4.8	0.68
Zung Depression Index	29.7	29.0	11.6	26.1	26.0	11.8	30.7	30.0	11.4	0.06
MSPQ Questionnaire	9.7	9.0	6.8	8.8	8.0	6.4	9.9	9.0	7.0	0.49
% Male	53.0			47.1			54.7			0.55
% Smoker	34.4			35.3			34.2			0.98
% Off work	50.0			44.1			51.7			0.33
% Previous spinal surgery	28.8			17.7			28.2			0.14
% traumatic onset	69.5			73.5			68.4			0.78

Notes: 1. p value refers to comparisons between included and excluded patients

* significant at p < 0.05

**Table 2:** Diagnostic value of clinical examination variables in relation to single anaesthetic zygapophysial joint block

**Reference Standard A (75% reduction in Pain VAS)**				
	
**N = 116^1^**		Contingency table		
		Injection result		
		Positive	Negative	
Criteria satisfied		3	8	
Criteria not satisfied		24	81	
**% Sn^3 ^**(95 CI^8^)	**% Sp^4 ^**(95 CI)	**% PPV^5 ^**(95 CI)	**% NPV^6 ^**(95 CI)	**+ LR^7 ^**(95 CI)
11.1 (3.9,28.1)	91.0 (83.3,95.4)	27.3 (9.8,56.6)	77.1 (68.2,84.1)	1.2 (0.3,4.3)
**Reference Standard B (Abolition of Primary Pain)**				
	
**N = 111^2^**		Contingency table		
		Injection result		
		Positive	Negative	
Criteria satisfied		3	6	
Criteria not satisfied		15	84	
**% Sn **(95 CI)	**% Sp **(95 CI)	**% PPV **(95 CI)	**% NPV **(95 CI)	**+ LR **(95 CI)
16.7 (5.8,39.2)	93.3 (86.2,96.9)	33.3 (12.1,64.6)	84.9 (76.5,90.6)	2.5 (0.7,9.1)

Notes: ^1^n = 116. One case with missing data items. Unable to determine satisfaction of criteria

^2^n = 111. Cases with available data on abolition of primary pain

^3^Sn = sensitivity, Sp^4 ^= specificity, PPV^5 ^= positive predictive value, NPV^6 ^= negative predictive value, +LR^7 ^= positive likelihood ratio, 95 CI^8 ^= 95% confidence interval
